# Synthesis of non-phosphorylated epoxidised corn oil as a novel green flame retardant thermoset resin

**DOI:** 10.1038/s41598-021-03274-z

**Published:** 2021-12-17

**Authors:** Maurelio Cabo, Prabhakar M. N., Jung-il Song

**Affiliations:** 1 Department of Smart Manufacturing Engineering, Changwon National University, Uichang-gu, Changwon, Gyeongsangnam-do 51140 Republic of Korea; 2 Research Institute of Mechatronics, Department of Mechanical Engineering, Changwon National University, Uichang-gu, Changwon, Gyeongsangnam-do 51140 Republic of Korea; 3 Department of Mechanical Engineering, Changwon National University, Uichang-gu, Changwon, Gyeongsangnam-do 51140 Republic of Korea

**Keywords:** Chemistry, Materials science

## Abstract

This study aimed to produce a new potential flame retardant thermoset resin from epoxidised corn oil through a one-pot method using liquid inorganic catalysed with hydrogen peroxide. Using a gas chromatography–mass selective detector, attenuated total reflectance-fourier transform infrared spectroscopy, proton nuclear magnetic resonance imaging, optical microscopy, and scanning emission microscopy, we synthesised a bio-based resin based on newly designed parameters. The flame retardant capacity was fully established using thermogravimetric analysis and a micro calorimeter. The produced epoxidised corn oil had a relative percentage conversion of oxirane of approximately 91.70%, wherein the amount of double bonds converted into epoxides was calculated. A significant reduction from 17 to 40% in peak heat rate release (pHRR) and 26–30% in total heat release was observed, confirming its flame retardant property. Thus, the potential of epoxidised corn oil was demonstrated.

## Introduction

Sustainable chemistry must involve highly efficient, safe, simple, practical, and low-toxicity processes. The exploitation of agro-based polymer products, such as vegetable oil, has led to the development of non-toxic and bio-based materials. The manufacturing of new polymeric materials from the utilisation of bio-based resources is a response to ecological and sustainability considerations. A bio-based product is a product synthesised from renewable resources, which is used for epoxy monomer synthesis, resulting in a reduction in environmental impacts such as non-renewable resource consumption^1^. The production of thermoset polymers has evolved in industrial applications. Hence, recent agro-based polymer products, such as vegetable oil, have gained popularity. The complexity of the chemical structure of vegetable oils is mainly attributed to the following components: triacylglycerol, diacylglycerols, free fatty acids, and other minor polar components^2^. Elucidating the characteristics of each component will provide an understanding of the differences in each component obtained from vegetable oil. Phospholipids, triglycerides, diglycerides, monoglycerides, and sterol esters can all be found in fatty acids^3^.

Vegetable oil is an important starting material for the oleochemical market because of its abundance, low price, and unique reactive chemical structure^4^. Two issues must be resolved before methods of the epoxidation of vegetable oil can be simplified: (1) the synthesis of thermosets without the presence of bisphenol-A (BPA) to address the environmental toxicity, and (2) the phosphorylation process for the reduction of the complex methods of producing flame retardant materials. Because some industrial countries, such as Canada, France, and Denmark, have declared BPA to be a toxic substance that poses risks to human health as well as to the environment, there is pressure to find its replacement in the immediate future^5,6^.

Epoxy monomers are commonly derived from the organic synthesis of bis(4-hydroxyphenylene)-2,2-propane (bisphenol-A), 1-chloroprene 2-oxide (epichlorohydrin), and sodium hydroxide. Moreover, the recent ban on BPA because of the negative impact of BPA on human health and the environment necessarily implies focusing research on its substitution^1^. The issue of eutrophication caused by phosphorylation, wherein the phosphorous enters the water and undergoes a phosphorous cycle, needs to be addressed^7^. The metric denotes compounds as high risk if they contain phosphorus (P), medium risk if they contain nitrogen (N), and low risk they contain neither N nor P^8^ In many commercial flame retardant compounds, phosphorus is known to be an effective and popular halogen-free material^9^ Most epoxidation processes involving vegetable oils and thermoset resins can proceed with effective flame retardancy; however, they risk the environmental effect of phosphorus by infusing phosphorylation into their methodologies^10–13^.

In this respect, much effort has been expended to produce bio-based epoxy thermosets manufactured from renewable resources' aliphatic epoxy monomers, with the critical impart of flame retardant characteristic, which is obtained directly from fatty acid^14^. Lligadas et al. developed a phosphorus-containing fatty acid-based epoxy monomer (DOPO-III) that was then cured with DDM and bis(m-aminophenyl) methyl-phosphine oxide (BAMPO)^11^. Tarik et al. used a one-step method to manufacture and polymerize bromoacrylated soybean and sunflower oil, with soybean oil providing a greater substitute than sunflower oil. The ignition response index (IRI) of the cured polymers was observed after copolymerizing with styrene^15^. Wang et al. studied a castor oil-based bio-mercaptan infused with butanedioic acid (DDP) and allyl glycidyl ether (AGE) to improve its flame retardant ability, which was produced on the wood surface by the thiol-ene click reaction^16^. Das et al. looked into flame retardant epoxy/clay nanocomposites made from vegetable oil utilizing mechanical shearing and ultrasonication. Over the virgin polymer, the nanocomposites showed a significant increase in performance aspects such as curing, mechanical, thermal, and chemical resistance^17^.

However, depending on the reactants and catalysts used, the methods and process of exploiting vegetable oils’ fatty acids into bio-based epoxy thermosets resin like epoxidation still continuously vary. Alkenes and other unsaturated hydrocarbon chains using epoxidation are one of the most essential organic synthesis processes, where the epoxide group is transformed into an intermediate. Furthermore, through chemical and enzyme treatment procedures, renewable resources, such as fats and oils, can be converted into materials that act as a replacement for materials derived from petroleum^18^. The available epoxidation methods are as follows: (a) epoxidation in photoinitiated cationic polymerization^19^, (b) epoxidation with percarboxylic acids^20,21^, (c) epoxidation with organic and inorganic peroxides^22^, (d) epoxidation with halohydrines^20^, (e) epoxidation with molecular oxygen^20^, and (f) epoxidation by thermal or UV-cationic processes^23^.

From the previously mentioned methods, epoxidation with percarboxylic acids and organic and inorganic peroxides needs to be developed. In fact, with respect to environmental and economic considerations, hydrogen peroxide is considered to be the best terminal oxidant after dioxygen^24,25^. With the recent concerns regarding global environmental problems, researchers have expounded their interest in developing products tailored from renewable and eco-friendly materials^26^. Epoxidation systems in conjunction with a liquid inorganic acid, sulfuric acid, as a catalyst have already been established as potentially viable for production, development, process, and continuous research on finding new bio-based polymers from agro-polymer products such as vegetable oil.

Corn is one of the most abundant commodity crops, contains 55% linoleic acid and 28% oleic acid, and can be used as a platform for new chemicals such as bio-based resins^27,28^. The advantages of this oil are as follows: (1) an abundant supply, (2) an extremely attractive price, approximately 1/4th that of soybean oil, and (3) a favourable fatty acid composition. In fact, converting corn oil to valuable chemicals and different polymeric products such as coatings, adhesives, elastomers, foams, plasticisers, and epoxy resins has been studied by the Kansas Polymer Research Center^29^. However, there is still limited related research on using its greater potential as a resource of cheap and environmentally friendly bio-based materials.

The technology applied involved the functionalisation of corn oil through epoxidation and ozonolysis, followed by different chemical processes to obtain starting materials for a wide range of industrial and household products. Producing these products from renewable resources will help reduce the world’s dependence on imported petroleum and create new markets^30^. Accordingly, bio-based compounds have recently gained from both academic and industrial perspectives, resulting in a new and strong interest in the development of epoxy cross-linking polymers with strong flame retardant properties. Thus, to address the problem of producing polymeric materials such as thermoset resins and flame retardant additives from toxic chemicals, this study explored, validated, and added value to products using *Zea mays* (corn) oil, a locally renewable resource, as a source of bio-based thermoset resins with flame retardant properties without resorting to phosphorylation.

## Experimental

### Materials

Corn oil was purchased from Sigma-Aldrich, Korea. By turbidity, it’s clear in appearance and yellow in color. Per supplier disclosure: Acid value at ≤ 0.2, peroxide at ≤ 10.0, and water content at ≤ 0.1%. 13 Fatty acids were detected which as follows: C14, C16, C16:1, C18, C18:1, C18:2, C18:3, C20, C20:1, C22, C22:1, and C24. Since no Iodine value was disclosed by the supplier, per our computation, it has 89.32 based on the Iodine value determination of ASTM D5554. Glacial acetic acid (99.7% v/v), sulfuric acid (98% v/v), hydrogen peroxide (34.5%, v/v), diethyl ether (99%, v/v), sodium hydroxide, Wijs reagent, sodium thiosulfate, cyclohexane (99% v/v), potassium iodide (99.5% v/v), potassium acid phthalate ( 99.95%), crystal violet, and hydrobromic acid (47–49% v/v) were purchased from Samchun, Korea. The KRF-1031 (bisphenol-A epoxy) Vinyl ester (viscosity = 150 cps and specific gravity = 1.03), cobalt naphthalate (accelerator), and methyl ethyl ketone peroxide (hardener) were produced by CCP Composites, Korea, while YD-128 (Digylcidyl ether of Bisphenol A) epoxy resins and methyl tetra hydropthalic anhydride (MTHA) were procured from Kukdo Chemicals. These and other materials applied in this study were used as received. All methods were performed in accordance with the relevant guidelines and regulations.

### Experimental setup

The epoxidation reactions were carried out in an erlenmeyer flask (500 mL) set up using magnetic stirrer over heating plate. Using constant volume of corn oil, 50 mL and set stirring speed for about 700–1200 rpm.

For thermoset’s preparation, 100 g of thermoset resins were mixed with adequate curing agent to make the thermoset. For epoxy resin, a 5:4 ratio of resin to curing agent (MTHA) was employed, but for ECO and Vinyl Ester, a curing agent of 1% MEKP and 1% accelerator was used. The mixes were placed in a curing oven with the following settings: 80 °C curing temperature for 6 h, and 120 °C post-curing temperature for 2 h, using a steel mold.

### Epoxidation procedure

Table 2 lists the reactive conditions used for epoxidised corn oil (ECO). It was prepared using acetic acid and aqueous H_2_O_2_ solution in the presence of a catalytic amount of sulfuric acid. The calculated amount of carboxylic acid and a liquid inorganic acid catalyst (1% of the total solution weight) was added to the vessel, and the mixture was stirred for approximately half an hour. Then, the required amount of 34.5% aqueous H2O2 was added drop-wise over half an hour, then the reaction was further continued for the desired time duration. In a separating funnel, the extraction process of the collected samples was carried out using diethyl ether. The product was successively washed with cold water and slightly hot water until the free acids were completely removed^31^. And for safety purposes, in this work, to decrease the danger of explosion, hydrogen peroxide at 34.5 wt% was used instead of the more usually employed 50 wt%. To minimize corrosion of the reactor, formic acid was substituted with acetic acid, Vianello et al. investigated the thermal behavior of peracetic acid^32–34^.

**Figure 1:**
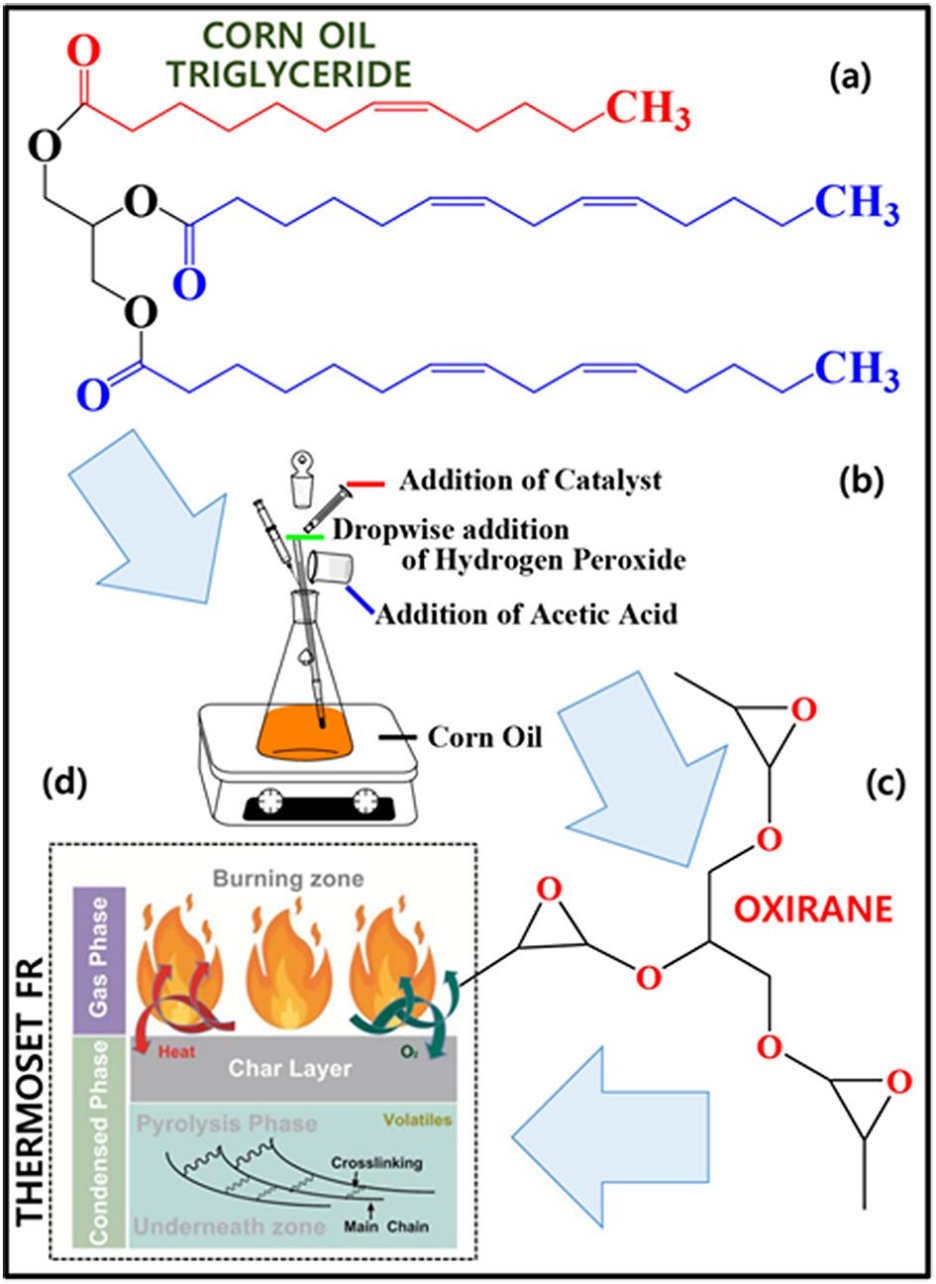
The synthesis route of the conversion of corn oil into epoxidized (**a**–**c**); and (**d**) proposed schematic diagram of the flame retardant mechanism.

Two technological parameters were used based on Dinda^31^ and Musik^35^ to optimise the molar ratio of H_2_O_2_ and double bonds. Because the relative oxirane percentage was below par, this study designed new parameters, 10:1 (H_2_O_2_:double bonds), which resulted in comparable end-product results as reported also by Vianello et al.^36^. According to their analytical results, epoxidised oil has a white colour and is very viscous, which were achieved in this study after increasing the amount of H_2_O_2_ to 10 molar ratio. Using orthogonal design, L_9_(3^4^), the results further assessed which variables affected epoxidation the most. The overall synthesis route of the conversion of corn oil into epoxidized and proposed schematic diagram of its flame retardant mechanism presented in Fig. 1.

### Analytical technique

Using wet lab analysis and conventional methods, iodine values were determined according to the Wijs method (ASTM D5554-95)^37^.

1 $$Iodine\;Number = \frac{{\left( {B - S} \right) \times N \times 12.69}}{{{\text{weight}}\;{\text{of}}\;{\text{sample}}}}$$

where B = titration of blank; S = titration of sample; and N = Normality of sodium thiosulphate solution.

Using hydrobromic acid solution in glacial acetic acid, the percentage of oxirane oxygen was determined using the direct method. With the use of potassium iodide, the iodic acid and periodate excess formed were reduced, and the liberated iodine was titrated with sodium thiosulfate. From the oxirane content values, the relative percentage conversion to oxirane (RCO) was calculated using the expressions provided by Paquot^38^.

2 $$Relative\;\%\; conversion\;to\;oxirane = \left( {\frac{{OO_{exp} }}{{OO_{the} }}} \right) \times 100$$

where OO_exp_ = oxirane oxygen experimentally and OO_the_ = oxirane oxygen theoretically.

3 $$OO_{{{\text{the}}}} = \frac{{\left\{ {\left( {{\text{IV}}_{{\text{o}}} /2{\text{A}}_{{1}} } \right)} \right\}{\text{A}}_{{\text{o}}} }}{{\left\{ {100 + \left( {{\text{IV}}_{{\text{o}}} /2{\text{A}}_{{1}} } \right){\text{A}}_{{\text{o}}} { }} \right\}}} \times 100$$

where A_l_ = 126.9 and A_o_ = 16.0, are the atomic weights of Iodine and Oxygen respectively while IV_o_ is the initial iodine value of the sample.

### Statistical analyses

Orthogonal design was used to determine suitable tolerances for the components of a certain assembly^39^. All experiments were performed at least in triplicate. Results were expressed as means ± Standard Errors of the Means (SEM). For orthogonal design analysis of experiments, a two-way analysis of variance (ANOVA) was used^40^. Statistical significance was considered at p < 0.05 thus, the following equations were used for this assessment:

4 $$K_{1} = \Sigma \;the\;amount\;of\;RCO\;at\;certain\;variable$$

5 $$k_{1} = K_{1} /3$$

6 $$R_{1} = \max k_{1} - \min k_{1}$$

where K is the sum of the all RCO values in each level per variable, k is the mean value of K, and R is the difference between the maximum and minimum mean value of k.

## Characterisation

### GC-MSD

The presence of double bonds among free fatty acids was detected using a gas-chromatograph-mass selective detector (Perkin Elmer, Korea) (Model number: Claus 690/SQ8), with a performance in the mass range of 1–1200 amu and an ionisation source of El (70 eV).

### ATR-FTIR

IR spectra were recorded on a Fourier-transform infrared spectroscopy (FTIR) spectrometer (FT-IR-6300, JASCO, United Kingdom) under dry air at ambient temperature. The percentage of transmittance spectra was recorded from 4000 to 400 cm^−1^ with 32 scans in each case at a resolution of 4 cm^−1^. Furthermore, using the principles of light and waves, the following equations related to these were used:

7 $$c = \nu \lambda$$

where c is the speed of light in centimetres per second, v is the frequency in Hertz (s^−1^), and λ is the wavelength in centimetres.

8 $$W = 1/\lambda$$

where W is the wavenumber in cm^−1^, and λ is the wavelength in centimetres.

9 $$E = hv$$

where E is the energy, h is Planck’ s constant, which equals 6.626 × 10–34 Js, and v is the frequency.

For crosslinking characterization, where the extent of epoxy reactions were determined by the peak areas of the oxirane peaks at 829 cm^−1^ (VE), 831 cm^−1^(EP), 875 cm^−1^ (ECO) in reference to the peak at 1033 cm^−1^ (VE), 1036 cm^−1^ (EP), 1162 cm^−1^ (ECO), which is due to C–O stretching, the below equation was followed^41,42^:

10 $$\alpha = 1 - \frac{{\left[ {\left( {A_{ot} - A_{co0} } \right)} \right]}}{{\left[ {\left( {A_{o0} - A_{cot} } \right)} \right]}}$$

where A_co0_ and A_cot_ refer to the areas of the reference peak at the time zero (0) or uncured and after curing time (t), respectively. A_o0_ and A_ot_ are the areas of oxirane peaks for uncrosslinked resin to crosslinked resin after curing time, respectively.

### ^1^H-NMR

The molecular interaction information was examined using nuclear magnetic resonance (NMR) machine (Bruker Avance, Korea) (Model number: 400 UltraShield) composed of a 400-MHz magnet, console box, Topshim systems, UPS, air dryer, and compressor. It provides information on the environment around the nucleus and spin bonds with neighbouring atoms due to energy absorption in the magnetic field of an atomic nucleus.

### Thermogravimetric analysis

The thermal stabilities of the three types of resins were characterised using a thermogravimetric analyser (TGA; Perkin Elmer STA 6000, England) within a temperature range of 30 to 650 °C at a rate of 20 °C/min under a nitrogen atmosphere.

### Optical microscopy and scanning electron microscopy

To study the surface morphology, the specimens were tested under an optical microscope (OM; Olympus, U MSSP4 model with Tech Xcam-III, Techsan Company Limited, Japan) and scanning electron microscope (SEM) at 20 kV (Model: Emcrafts cube 2, EMCRAFTS.CO, Korea). The specimens were sputter-coated with gold using an auto fine coater (JEOL JFC-1600).

### Micro-calorimetry

The flammability properties of the samples were examined using a micro calorimeter (Model Number: SG-5300) test apparatus, FAA Micro Calorimeter (Federal Aviation Administration, Fire Testing Technology (FTT), UK), to calculate the heat release rate (HRR) and total heat release rate (THR) according to the ASTM D 7309 standards. It is a low-cost tool for screening and predicting the flammability of polymers and other materials. In this flammability test technique, during pyrolysis, the gases are released into an oven at 900 °C containing an 80:20 mixture of N_2_:O_2_.

## Results and discussion

### Free fatty acid analysis

**Figure 2:**
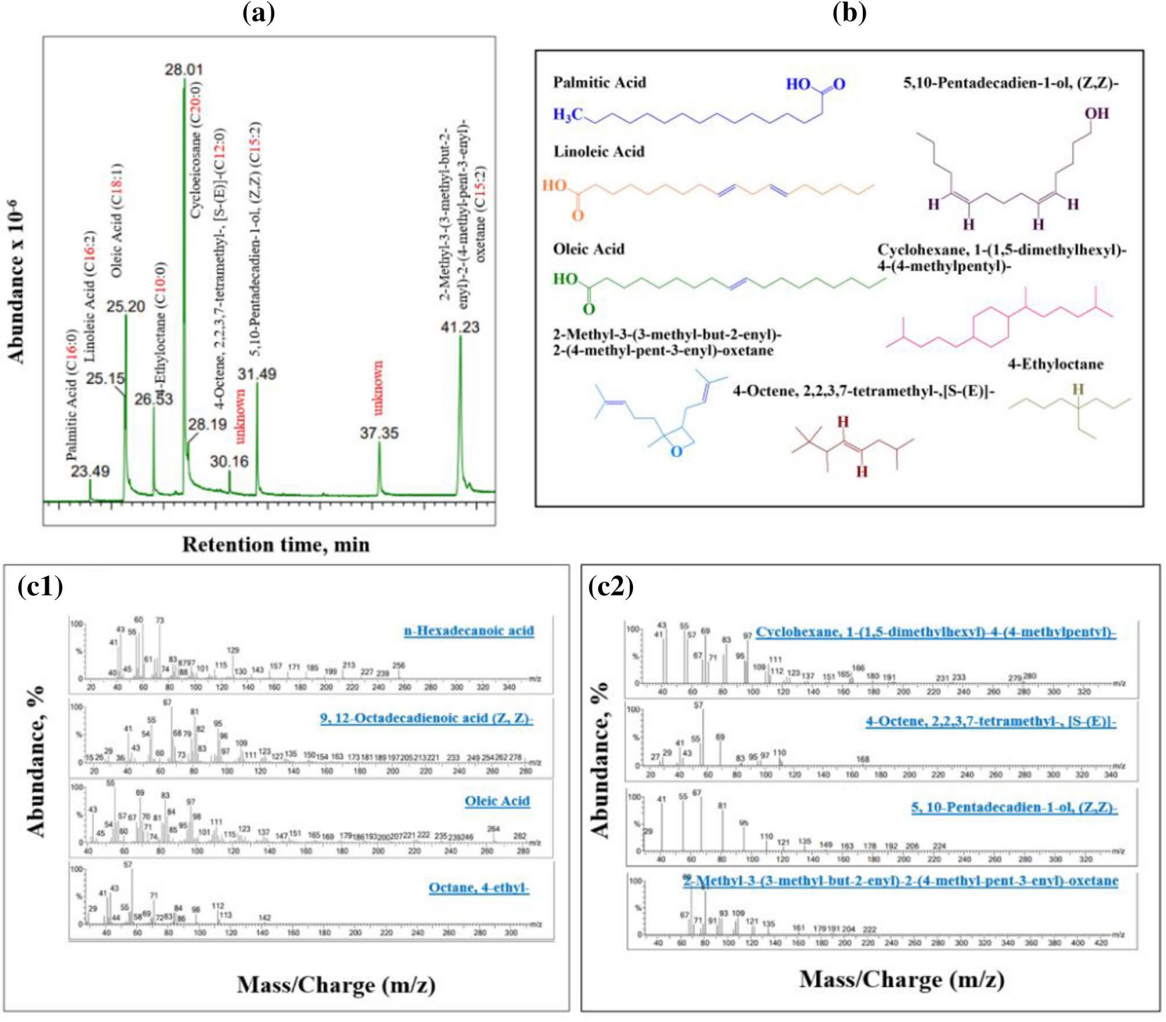
Free fatty analysis using gas chromatography–mass spectrometry detector for the unepoxidised corn oil: (**a**) GC–MS report confirming the double bonds; (**b**) chemical structure of 8 detected free fatty acids; and (**c1** and **c2**) mass/charge (m/z) vs abundance report.

The specific fatty acids were determined through direct polar stationary phases using gas chromatography–mass spectrometry^43^. An easier and quicker sample preparation using the free form method was used to avoid the possible alteration of epoxides formed, instead of methyl esters^44^. The results showed that for unepoxidised corn oil, eight free fatty acids were detected. Categorically, four were saturated or lacked double bonds, three were monounsaturated or containing single double bonds, and one was polyunsaturated or containing two double bonds (see Table 1). Figure 2a shows the chromatogram of the sample, where the main components were identified, completely separated, and eluted from each other. The chemical structures of the detected components are shown in Fig. 2b.

**Table 1:** IUPAC name and its molecular weight of the detected free fatty acids.

FFA	IUPAC name	Common name	Molecular weight	Double bonds
C16:0	n-Hexadecanoic Acid	Palmitic Acid	256.0	Saturated
C16:2	9,12-Octadecadienoic acid (Z,Z)	Linoleic Acid	280.0	Polyunsaturated
C18:1	Oleic Acid	Oleic Acid	282.0	Monounsaturated
C10:0	Octane, 4-ethyl-	4-Ethyloctane	142.0	Saturated
C20:0	Cyclohexane, 1-(1,5-dimethylhexyl)-4-(4-methylpentyl)-	–	280.0	Saturated
C12:0	4-Octene, 2,2,3,7-tetramethyl-, [S-(E)]-	–	168.0	Saturated
C15:1	5,10-Pentadecadien-1-ol, (Z,Z)-	–	224.0	Monounsaturated
C15:1	2-Methyl-3-(3-methyl-but-2-enyl)-2-(4-methyl-pent-3-enyl)- oxetane	–	222.0	Monounsaturated

Palmitic acid (C16:0) appeared at a retention time of 23.49 min followed by linoleic acid (C18:2), which appeared at a retention time of 25.15 min. Oleic Acid (C18:1), 4-ethyloctane (C10:0), cyclohexane, 1-(1,5-dimethylhexyl)-4-(4-methylpentyl)- (C20:0), and 4-octene, 2,2,3,7-tetramethyl-,[S-(E)]-(C12:0) peaks appeared at a retention time of 26.53, 28.01, and 28.19 min, respectively. Finally, 5,10-pentadecadein-1-ol,(Z-Z)-(C15:1), and 2-methyl-3-(3-methyl-but-2-enyl)-2-(4-methyl-pent-3-enyl)-oxetane (C15:1) appeared at retention times of 31.49 and 41.23 min, respectively.

Two unknown peaks were observed, which did not match the corresponding list provided for mass spectrometry. Out of the four compounds detected, which have no common name, two exhibited double bonds. Interestingly, 5,10-pentadecadein-1-ol,(Z-Z)- (C15:2) and 2-methyl-3-(3-methyl-but-2-enyl)-2-(4-methyl-pent-3-enyl)-oxetane might suggest that the unepoxidised corn oil contained unpublished compounds, which might help to progress additional future research on converting all double bonds into epoxy, regardless of their abundance. Furthermore, this idea is supported by their molecular weight, as determined using mass spectrometry, which were 224 and 222 m/z, respectively, as reported in Table 1. These values are not too far from linoleic acid (280 m/z) and oleic acid (282 m/z). All components were verified using MS and are presented in Fig. 2c1, c2.

Additionally, as per the disclosure of fatty acids content in the product specifications mentioned in “Materials” section, four (4) fatty acids confirmed by GC–MS via free fatty acid analysis namely: C16, C18:1, C18:2, and C20.

### Wet lab analysis

The capacity of corn oil to produce epoxides was investigated using orthogonal design, a design of optimum multifactorial experiments using three levels and four variables, L_9_(3^4^) following the nine entries of the experiment, see Table 2. Additional two entries in the experiment were added, entry 10 and 11 following the entry 1 design comprising all variables in the same level. The two established technological parameters and reactive conditions were used based on the epoxidation synthesis processes of Dinda et al.^31^ and Musik et al.^35^ for level 1 and level 2, respectively. Level 3 was this research newly offered parameters and reactive conditions.

**Table 2:** Processing parameters for the epoxidation of vegetable oils using homogeneous system in orthogonal design.

Entry	Catalyst (%w/w)	Oxidant	Reaction conditions	DB: H2O2: Acid	Iodine value (per 100 g)	Relative % conversion of Oxirane (RCO)
1^31^	H_2_SO_4_/H_2_O_2_ + CH_3_COOH	Peracetic acid	60 °C, 4 h, 850 rpm	1: 1.5: 0.5	50.21 ± 1.91	33.41 ± 2.75
2			60 °C, 4 h, 700 rpm	1: 3.5: 0.8	84.64 ± 1.52	16.04 ± 1.78
3			60 °C, 4 h, 1200 rpm	1: 10: 0.9	25.15 ± 2.70	90.46 ± 4.46
4			90 °C, 4 h, 850 rpm	1: 3.5: 0.9	54.71 ± 1.90	25.81 ± 1.86
5			90 °C, 4 h, 700 rpm	1: 10: 0.5	30.08 ± 4.23	40.13 ± 2.03
6			90 °C, 4 h, 1200 rpm	1: 1.5: 0.8	57.97 ± 6.39	20.50 ± 2.67
7			50 °C, 4 h, 850 rpm	1: 10: 0.8	84.99 ± 5.24	16.11 ± 5.87
8			50 °C, 4 h, 700 rpm	1: 1.5: 0.9	86.47 ± 1.52	16.39 ± 2.18
9			50 °C, 4 h, 1200 rpm	1: 3.5: 0.5	74.80 ± 0.01	13.32 ± 0.80
10^35^			90 °C, 4 h, 700 rpm	1: 3.5: 0.8	33.82 ± 4.59	42.56 ± 3.66
11			50 °C, 4 h, 1200 rpm	1: 10: 0.9	25.12 ± 2.08	91.70 ± 4.53
Variable A—Temperature: (Level 1) 60 °C; (Level 2) 90 °C; (Level 3) 50 °C Variable B—Stirring Speed: (Level 1) 850 rpm; (Level 2) 700 rpm; (Level 3)1200 Variable C—Hydrogen Peroxide Molar Content (H_2_O_2_): (Level 1) 1.5; (Level 2) 3.5; ( Level 3) 10 Variable D—Acetic Acid Molar Content: (Level 1) 0.5; (Level 2) 0.8; (Level 3) 0.9						
*K*_*1*_* : 139.91 (A); 75.33 (B); 70.30 (C); 86.86 (D)* *K*_*2*_* : 86.44 (A); 72.56 (B); 55.17 (C); 52.65 (D)* *K*_*3*_* : 45.82 (A); 124.28 (B); 146.70 (C); 132.66 (D)* *k*_*1*_* : 46.64 (A); 25.11 (B); 23.43 (C); 28.95 (D)* *k*_*2*_* : 28.81 (A); 24.19 (B); 18.39 (C); 17.55 (D)* *k*_*3*_* : 15.27 (A); 41.43 (B); 48.90 (C); 44.22 (D)* *R : 31.36 (A); 17.24 (B); 30.51 (C); 26.67 (D)*						

The determination of the iodine value of epoxidized corn oil using Dinda et al. of optimal reactive conditions yielded 50.21 ± 1.91 (entry 1), and using Musik et al. optimal process resulted, 33.82 ± 4.59 (entry 10). For the relative percent conversion of oxirane, entry 1 resulted in 33.41% ± 2.75 conversion and entry 10 yielded 42.56% ± 3.66. Thus, these established parameters showed not favourable to epoxidize corn oil due to low relative percentage conversion of oxirane.

A more orthogonal examination was required, and the *K, k,* and *R* values were computed and shown in Table 2. According to the *R* values, the most important effect on epoxidation was determined to be temperature. The effect of epoxidized corn oil on the ROC reduced in the following order:temperature > H_2_O_2_ molar content > acetic acid molar content > stirring speed.

Following the constant conditions of the percentage of catalyst and time of epoxidation, the influence of temperature followed by hydrogen peroxide as an efficient oxidant, might be caused by the intermolecular interaction of additional hydroxyl groups from hydrogen peroxide to co-polymerise as free radicals into triglyceride molecules. Likewise, the long-chain hydrocarbons, which are mostly unsaturated, provide additional opportunities for chemical modifications of plant-oil-based macromolecules; thus, there is a high possibility of developing new types of bio-based polymers. In addition, two reactive sites are present in an agro-based polymer product such as corn oil: a vinyl double bond, CH_2_=CH–C(O), which yields chain propagation and isolated double bonds in fatty acid isomers, and CH_2_–CH=CH–CH_2_, which contains allylic hydrogen atoms following the degradation chain transfer caused by the formation of less active radicals. For this reason, free-radical chain copolymerisation is an efficient method for this synthesis to tailor the desired properties^45^.

According to the *K* value, the optimal epoxidation variables were A_1_B_3_C_3_D_3_ (60 °C, 1200 rpm, 10:1 mol ratio of H_2_O_2_ to double bonds, and 0.90:1.0 molar ratio of acetic acid to double bonds). These optimal conditions, almost similar to entry 11 which yield 91.70%. In order to reduce the cost of production and maintain the activity of catalyst at the lower temperature, we selected the optimum technology of entry 11 which as follows: 50 °C, 1200 rpm, 1:10:0.9 mol ratio of double bonds/H_2_O_2_/acetic acid, and 4 h. A comparable result of percent conversion of oxirane was obtained as disclosed in published review articles from Wai et al.^46^ and Baroncini^47^. The latter result is much higher than the reported conversion percentages of sesame oil^45^, rice bran oil^48^, and cottonseed oil^31^.

Overall, the newly designed technological parameters for the epoxidation of corn oil yield sufficient relative percentage conversion of oxirane to offer a new bio-based polymeric material as a potential thermoset resin.

### Spectral analysis

**Figure 3:**
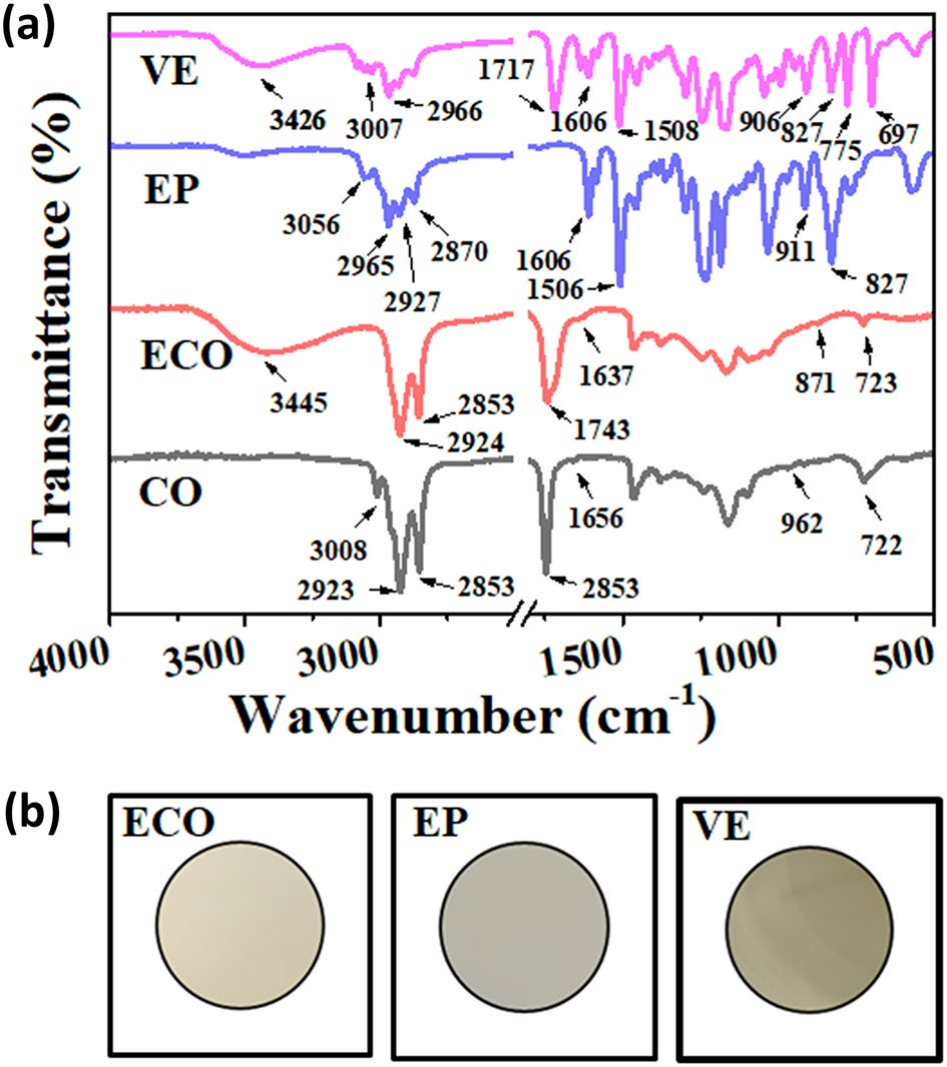
(**a**) Overall spectra of vinyl ester, epoxy, and epoxidised corn oil (ECO), and corn oil (neat); and (**b**) Digital images (top view) of two commercial thermoset resins and epoxidised corn oil.

Figure 3 shows the comparative IR analysis and digital images of two commercial thermoset resins (vinyl ester and epoxy resin) and the bio-based resin produced from ECO. The detailed assignments of the absorption bands are listed in Table 3. From the overall spectra in Fig. 3a, significant changes in band stretching were observed in the hydroxyl, ester, and oxirane groups. The disappearance of the absorption band at 3008 cm^−1^ in corn oil after epoxidation and the appearance of new peaks at 903 and 871 cm^−1^ indicate the presence and conversion of unsaturated into oxirane^49,50^. This is comparable with the epoxy resin presence of oxirane at 911 cm^−1^ and the absence of absorption bands at 3000–3010 cm^−1^. In addition, the new strong intensity of ECO at 3445 cm^−1^ is attributed to the presence of O–H, which may be the reason for its flame retardant property. This peak is comparable to that of vinyl ester, which shows -OH stretching at 3426 cm^−1^ and an improvement with epoxy resin peaks at 3056 cm^−1^.

**Table 3:** FTIR absorption bands and its designation.

Samples	3200–2000 cm^−1^	Assignment	1800–1500 cm^−1^	Assignment	1000–600 cm^−1^	Assignment
Vinyl Ester	3426	Hydroxyl Stretching Region intermolecular bonded	1717	Carbonyl group of ester (C=O)	990	C=C bending, alkene, monosubstituted
	3082		1631	Stretching and bending of vinyl groups (C=C)	943	Methacrylate group peak
	3058		1606	C=C aromatic vibrations	906	Styrene double bonds
	3029		1579	C=C aromatic vibrations	827	Aromatic ring
	2966		1508	Aromatic ring	775	C–H bending, 1,2-substituted
	2930				697	Out of plane C–H vibration in mono-substituted aromatic rings
	2871					
	3007	C–H stretching, alkene				
Epoxy	3056	C–H stretching, alkene	1606	C=C aromatic vibrations, conjugated alkene	968	C=C bending alkene
	2997	CH stretching alkane	1581	Aromatic C=C bond of phenolic ring	911	Oxirane
	2965		1506	Aromatic C=C bond of phenolic ring	863	Para sub of phenolic ring
	2927				827	Aromatic ring
	2870				766	C–H bending, 1,2-substituted
	2755	C–H aldehyde			733	C–H bending, 1,2-substituted
					664	Out of plane C–H vibration in monosubstituted aromatic rings
ECO	3445	O–H stretching	1743	C=O symmetric	945	C=C bending alkene, shift (displacement)
	2924	C–H stretching, alkane	1711	C=O in COOH group	903	Epoxide rings
	2853		1637	C=C stretching conjugated alkene	871	Epoxide rings
					767	C–H bending, 1,2-substituted
					723	Benzene derivatives
CO_Neat	3008	C–H stretching, alkene	1742	C=O symmetric	962	C=C bending alkene
	2952	C–H stretching, alkane	1656	C=C stretching conjugated alkene	722	Benzene derivatives
	2923					
	2853					

However, to evaluate the produced bio-based thermoset resin as a new material from corn oil, a comparison of absorption band assignments in the fingerprint region (1500–500 cm^−1^) was conducted. The appearance of a new peak at 767 cm^−1^ from ECO and the absence of peaks near 664 and 697 cm^−1^ observed from commercialised epoxies, indicating the possibility of confirming a newly produced bio-based resin. In the 1500–1800 cm^−1^ diagnostic region, ECO produced a new peak at 1711 cm^−1^, which is comparable to that of vinyl ester at 1717 cm^−1^.

**Figure 4:**
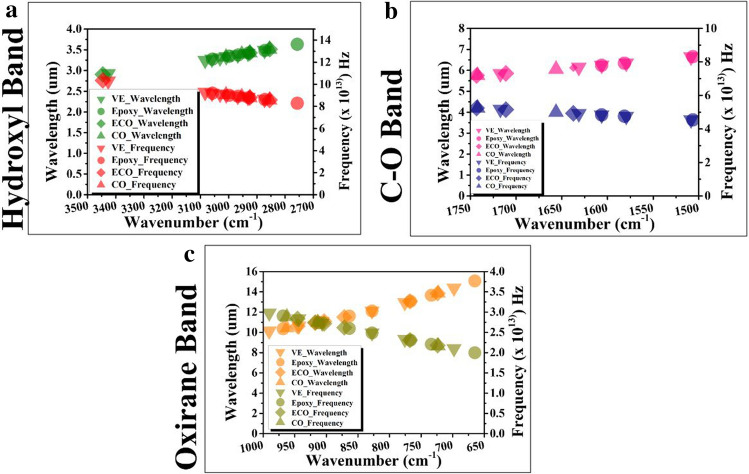
Behaviour of commercial thermoset resins, epoxidised corn oil and pure corn oil through light waves extracted from FTIR at the following adsorption bands: (**a**) hydroxyl band; (**b**) C-O band; and (**c**) oxirane band.

To further understand the spectra, the wavenumber was converted into wavelength and frequency, as shown in Fig. 4. Through the electromagnetic spectrum, this research would like to assess new materials from an additional perspective. Because every functional group is composed of different atoms and bond strengths, vibrations are unique to functional groups; thus, FTIR spectroscopy takes advantage of how IR light changes the dipole moments in molecules corresponding to specific vibrational energies for quantitative analysis^51^.

In the hydroxyl group, the frequency yields of the ECO, which was approximately 10.33 × 10^13^ Hz, is higher than that of vinyl ester’s frequency of 10.27 × 10^13^ Hz at an absorption band of approximately 3400 cm^−1^ as shown in Fig. 4a. This result is the same with the ester group, for which ECO produced a frequency as high as 5.84 × 10^13^ Hz (1711 cm^−1^), while the epoxy produced a frequency of 4.82 × 10^13^ Hz (1606 cm^−1^), and vinyl ester produced a frequency of 5.15 × 10^13^ Hz (1717 cm^−1^), Fig. 4b. However, in the oxirane group, Fig. 4c, ECO vibrated at a frequency of 2.71 × 10^13^ Hz, which is slightly less than the frequency value of epoxy at 2.73 × 10^13^ Hz. An increase in frequency also means an increase in photon energy, as mentioned in Planck’s law.

Thus, in terms of intermolecular movement, the hydroxyl and ester groups of ECO carry more energy, while in oxirane, much less energy than the commercial thermoset resins. The greater the amount of energy, the stronger the molecular bonding, and these results might affirm the developed bio-based material from corn oil as a new thermoset resin.

**Figure 5:**
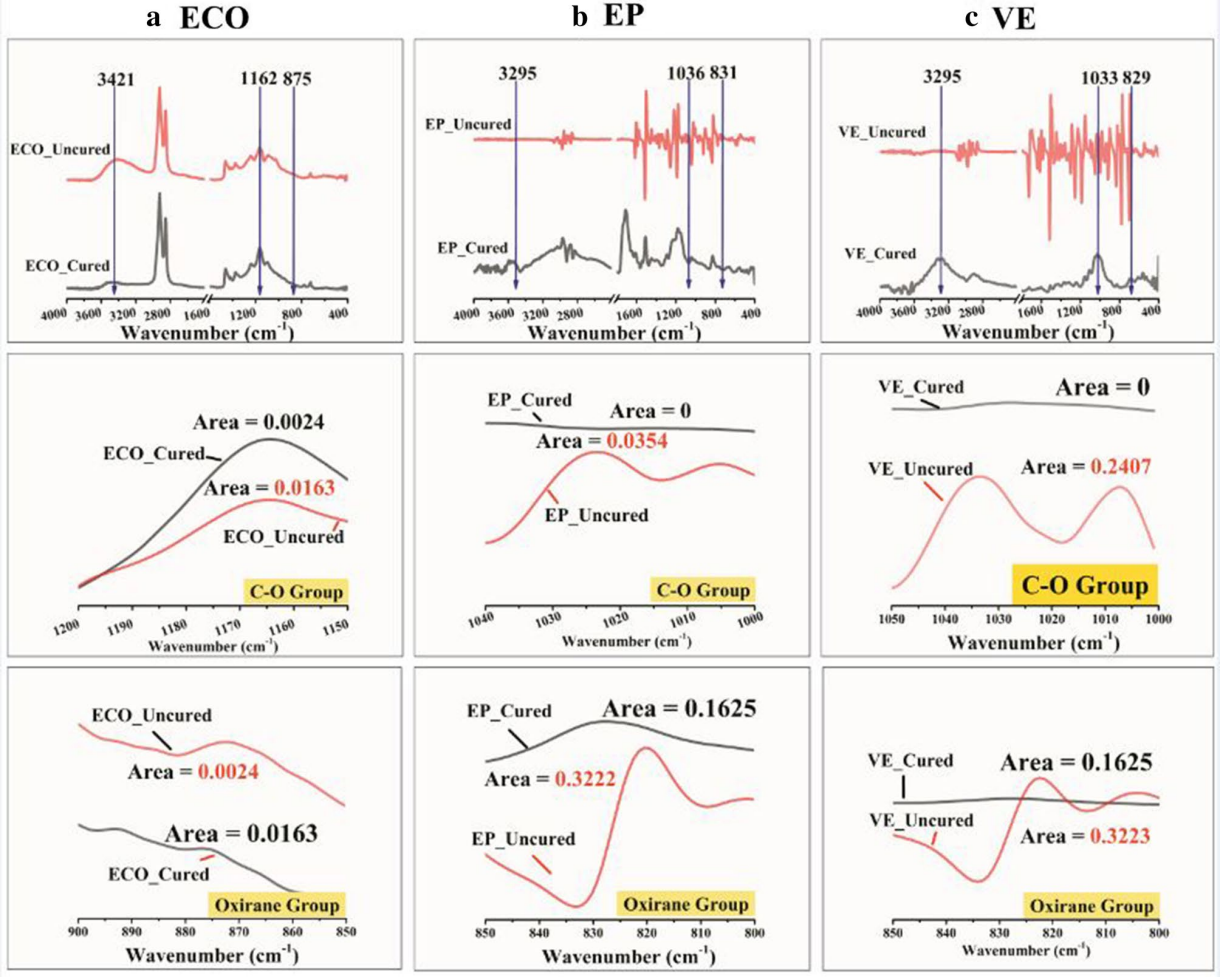
Crosslinking characterization by estimating epoxy conversion using FTIR spectroscopy for (**a**) epoxidized corn oil; (**b**) epoxy resin; (**c**) vinyl ester resin.

In Fig. 5, cured thermoset resins spectra also provided (C-O and oxirane band) to theoretically estimate epoxy group conversion. Following the crosslinking characterization by IR spectroscopy detailed by Nikolic et al.^41^ and peaks assignment studied by Gonzalez et al.^42^, significant peaks area are needed to calculate the epoxy conversion percentage. For ECO, C-O group peaks area are: 4.29 (uncured) and 2.73 (cured), in oxirane group peaks area: 0.0163 (uncured) and 0.0024 (cured), equivalent to 76.79% epoxy conversion; for EP, C-O group peaks are: 0.0354 (uncured) and 0 (cured), in oxirane group peaks area: 0.3222 (uncured) and 0.1625 (cured), equivalent to 100% epoxy conversion; for VE, C-O group peaks are: 0.2407 (uncured) and 0 (cured), in oxirane group peaks area: 0.3223 (uncured) and 0.1625 (cured), equivalent to 100% epoxy conversion. The disappearance of peak in C-O group and significant reduction of peaks assignment in oxirane group after curing might estimates into 100% epoxy conversion.

### ^1^H-NMR analysis

**Figure 6:**
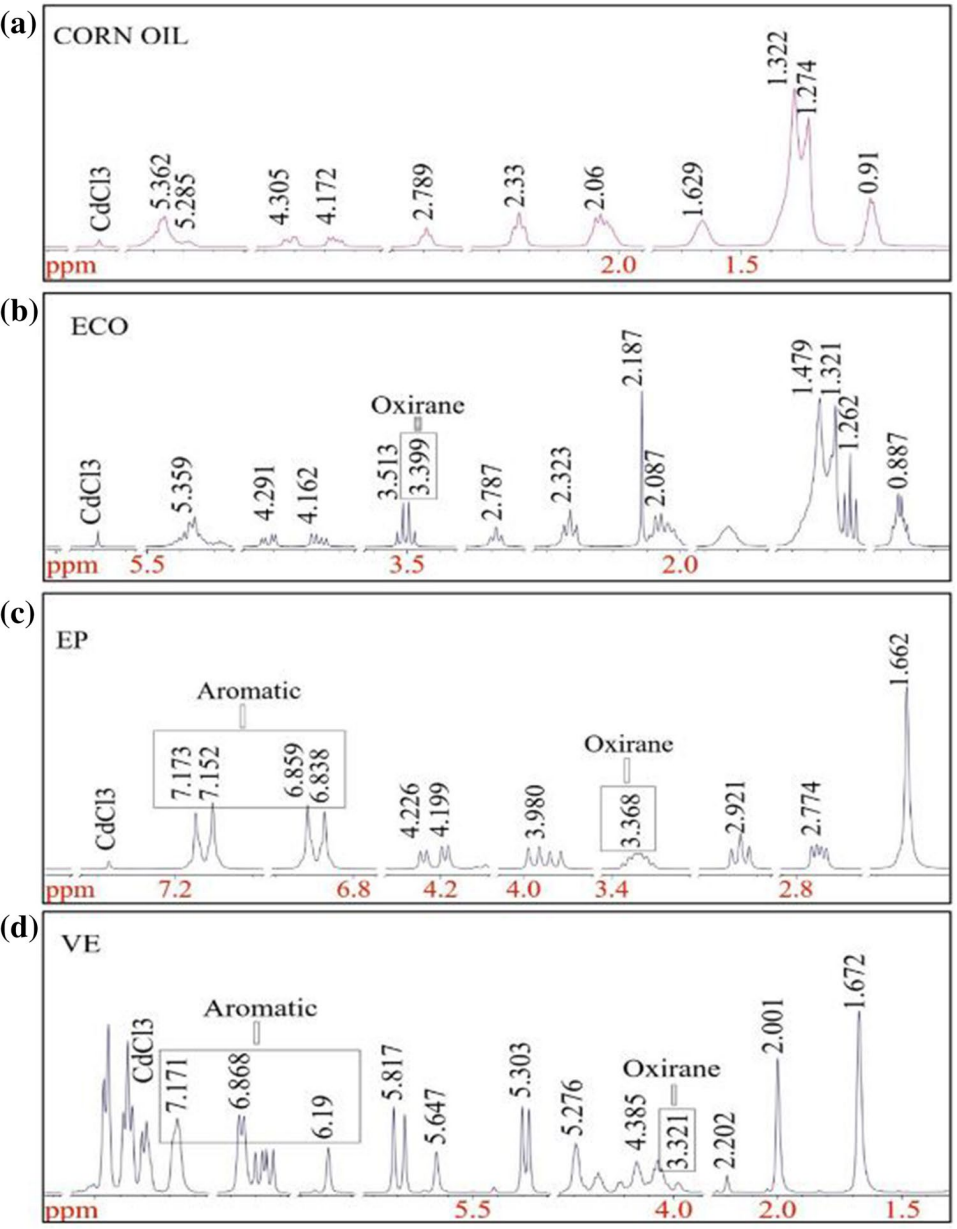
Proton NMR analysis for (**a**) corn oil_neat; (**b**) epoxidised corn oil; (**c**) epoxy resin; (**d**) vinyl ester resin.

The functionalisation of the ECO synthesis was also supported by ^1^H-NMR spectroscopy. The chemical shifts of ^1^H signals are affected by the proximity of electronegative atoms in the bonding network and by their proximity to unsaturated groups^49^. Based on the chemical shift values listed in Table 4 and shown in Fig. 6b, the presence of oxirane at δ 3.399 ppm for ECO confirms the conversion of unsaturation into epoxies, which supports the disappearance of signals at a chemical shift of δ 5.285 ppm from corn oil, see Fig. 6a, in the vinyl group^50^.

**Table 4:** Proton NMR chemical shift values in ppm.

	1°-Alkyl	2°-Alkyl	3°-Alkyl	Allylic	Carbonyl	Oxirane	Hydroxyl	Vinyllic	Aromatic
Corn Oil	δ 0.91	δ 1.274	δ 1.629	δ 2.06	δ 2.33		δ 4.172	δ 5.285	
		δ 1.322			δ 2.789		δ 4.305	δ 5.362	
ECO	δ 0.887	δ 1.262	δ 1.613	δ 2.087	δ 2.323	δ 3.399	δ 4.162	δ 5.393	
		δ 1.321		δ 2.187			δ 4.291		
		δ 1.479							
Epoxy Resin			δ 1.662		δ 2.774	δ 3.368			δ 6.859
					δ 2.921				
Vinyl Ester		δ 1.304	δ 1.672	δ 2.001	δ 2.202	δ 3.321	δ 4.063	δ 5.276	δ 6.19
							δ 4.385	δ 5.303	δ 6.73
								δ 5.647	δ 6.868
								δ 5.817	δ 7.171

The same chemical shifts were observed for the commercialised thermoset resins, which were approximately δ 3.368 ppm for epoxy resin, Fig. 6c and δ 3.321 ppm for vinyl ester, Fig. 6d, and no chemical shift was observed for neat corn oil, which strongly indicates the successful epoxidation process. However, the absence of aromatic rings in ECO might indicate high curing and brittleness properties because the bond energy and rigid structure of the aromatic ring present in commercialised thermoset resin helps to reduce the curing temperature^51^, while the presence of hydroxyl at δ 4.162 ppm and δ 4.291 ppm chemical shift values might be the basis for its flame retardant properties.

### Thermogravimetric analysis

**Figure 7:**
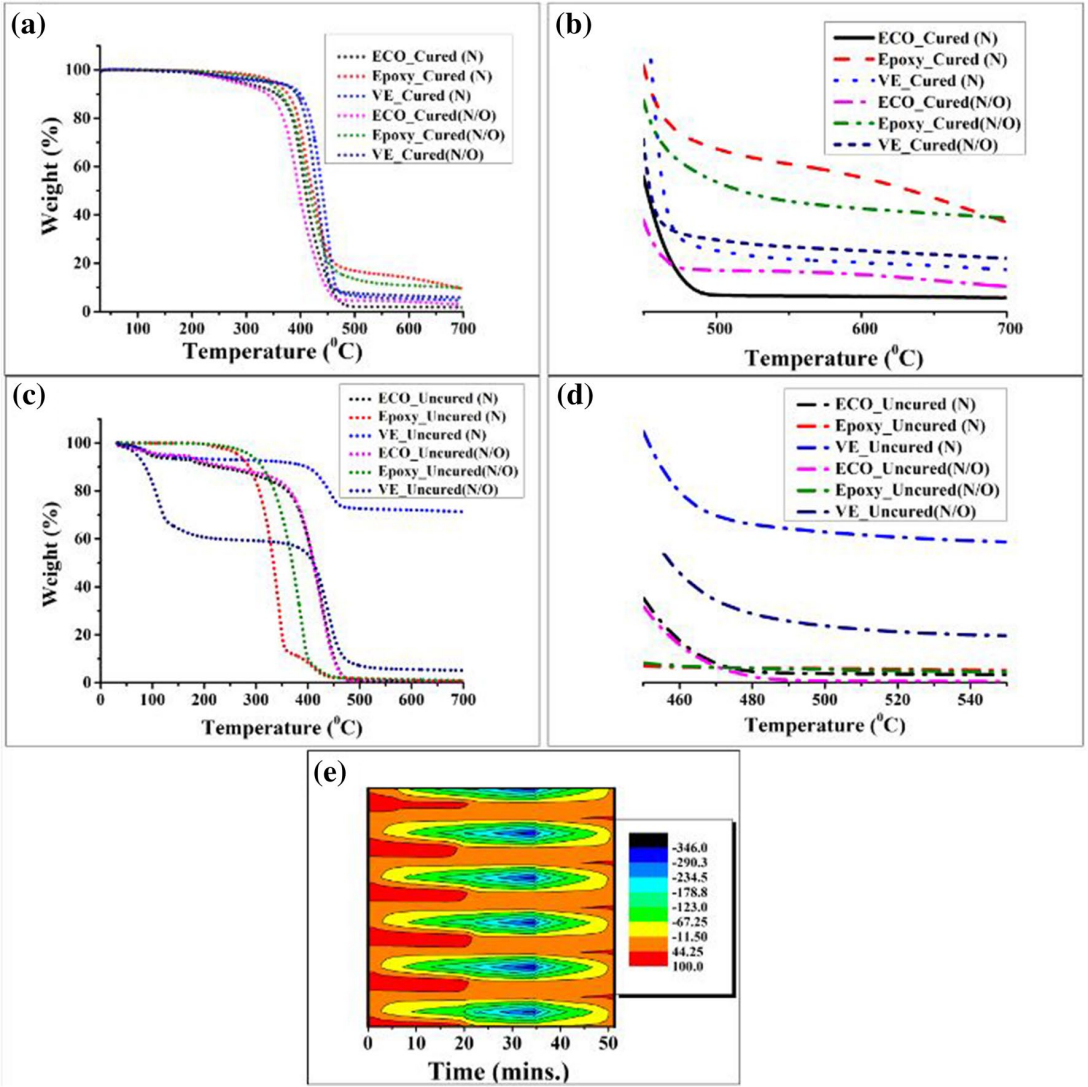
Thermogravimetric curves with residue magnification of the samples cured and uncured [(**a**) to (**d**)], while the heat flow influx was shown in (**e**).

The thermal stabilities of the uncured and cured thermoset resin samples were investigated using TGA. The ECO and vinyl ester (VE) were cured using methyl ethyl ketone peroxide (MEKP) as the hardener and cobalt naphthalate as the accelerator. The epoxy was cured using MTHA as a hardener. Figure 7 shows the TGA curves under nitrogen and a mixture of oxygen and nitrogen flow in air and the Table 5 discloses the thermal degradation at onset and maximum temperature and the percent residue produced after the analysis. Two main degradation stages were observed in all samples, whether cured or uncured. For the nitrogen flow, the temperature onset, temperature maximum, and the residue was at 700 °C were determined. The same settings for maximum and onset were used for the nitrogen and oxygen flow; however, the recorded residue was adjusted to 525 °C owing to the total degradation of ECO and epoxy at 700°C^52^.

**Table 5:** Thermogravimetric analysis test results thermoset resins samples.

Test name	Thermogravimetric analysis					
	Nitrogen			Air		
Sample name	T_onset_ (°C)	T_max_ (°C)	Residue at 700 (°C)	T_onset_ (°C)	T_max_ (°C)	Residue at 525 (°C)
ECO_Cured	364.76	456.65	1.42	347.28	446.36	4.16
Epoxy_Cured	376.86	463.03	9.12	358.00	458.97	11.97
VE_Cured	397.47	466.08	4.28	391.72	458.25	6.9
ECO_Uncured	360.61	468.94	0.2	361.36	461.52	0.19
Epoxy_Uncured	281.97	363.94	0.73	320.91	410.15	1.48
VE_Uncured	410.15	455.59	71.21	399.39	472.27	6.29

For the uncured sample in nitrogen flow, as shown in Fig. 7c, d, vinyl ester showed much higher thermal stability than the two samples at the 410.15 °C (onset) and at 455.59 °C (maximum), while the ECO followed at 360.61 °C (onset) and 468.94 °C (maximum), which showed a 27.89% (onset) and 28.85% (maximum) improvement over epoxy at approximately 281.97 °C (onset) and 363.94 °C (maximum). Under air flow for uncured samples, same trend follows: VE > ECO > EP. Meaning, VE and epoxy degrade much faster than ECO upon the addition of oxygen flow.

Upon the application of curing agents, the cured ECO exhibited still much lower thermal stability than the cured VE and EP thermoset resins (Fig. 7a, b) which now following the trend as VE > EP > ECO. This suggests that the curing agent used for ECO produced a lower cross-linking density than the other two samples^53^. Thus, even though the uncured ECO produced much higher thermal stability than uncured EP at the two thermal degradations, the residue of both cured and uncured ECO yielded much less, probably because of the presence of higher long-chain aliphatic compounds and the absence of aromatic rings, as confirmed using proton NMR. Furthermore, as disclosed in Fig. 5, the epoxy conversion using FTIR confirmed this hypothesis wherein only 76.79% of ECO yielded to be converted from oxirane yet 100% yield was theoretically observed for both VE and EP.

Finally, to illustrate a typical temperature profile under adiabatic conditions, a combustion wave through the simulation is shown in Fig. 7e. This is where the flux of the initial reactive mixture carries internal chemical energy that is released during the reaction and is converted into heat, which exchanges both matter and energy (heat) with the environment^54^.

This shows the relationship between the unit of heat expressed in watts (W) and the sample size in grams (g) upon application of heat up to 20 min across all the samples through changes in the flame colour, from red to green, which is due to combustion of carbon particles. The heat flow was approximately 40–100 °C. Over 20–50 min, the flame colour changed from yellow to blue-violet, which is indicative of the full combustion of carbon particles with or without the presence of oxygen. Thus, the heat flow ran from − 11.50 °C to − 346 °C.

### Surface morphologies

**Figure 8:**
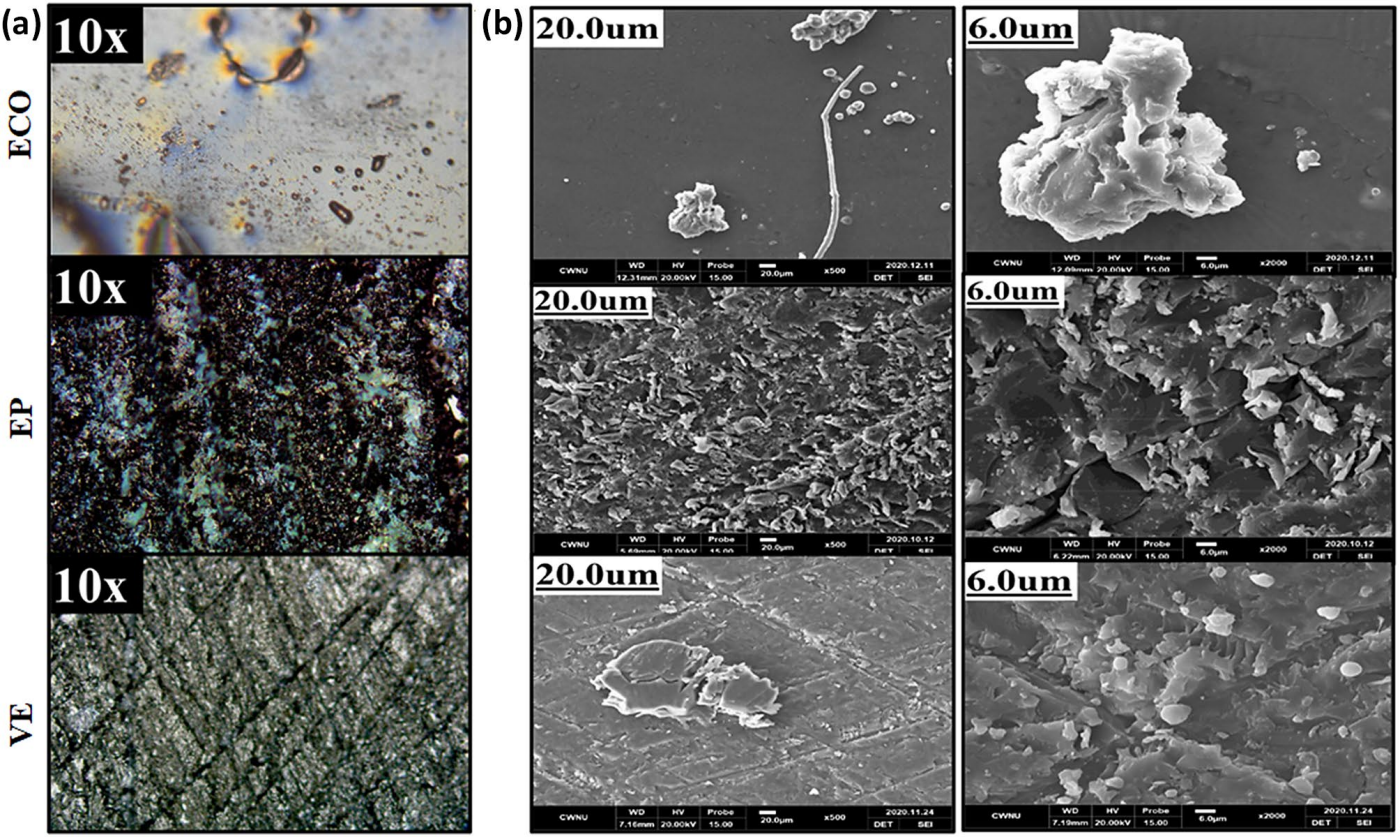
Images from (**a**) OM and (**b**) SEM for surface analysis of cured epoxidized corn oil; epoxy resin; and vinyl ester resin.

The OM image in Fig. 8a and SEM image in Fig. 8b reveal continuous and dispersed phases, resulting in rough surfaces for cured samples of ECO, epoxy, and vinyl ester. Although two different types of curing agents, anhydride for epoxy and organic peroxide for vinyl ester and ECO, were used, all thermoset resin samples were agglomerates with no determined size or shape. There appears to be a result of particle growth in random directions, which is consistent with the ATR-FTIR and ^1^H-NMR analyses that show that the product is not a pure compound^55^.

After curing, the cured ECO surface exhibited an almost smooth texture, as revealed through OM at 10 × magnification, while under SEM, the dispersed amorphous agglomerates showed prominent ridges at 6.0 µm magnification. Although epoxy and VE showed transparent morphology, they do not necessarily indicate homogeneity. The fracture morphology suggests brittleness and the result of the different reaction rates under the applied curing conditions^56^. Therefore, the differences between ECO and the two commercialised thermoset resins in terms of surface and fracture morphologies might confirm the derivation of new materials from corn oil.

### Microcalorimeter

**Figure 9:**
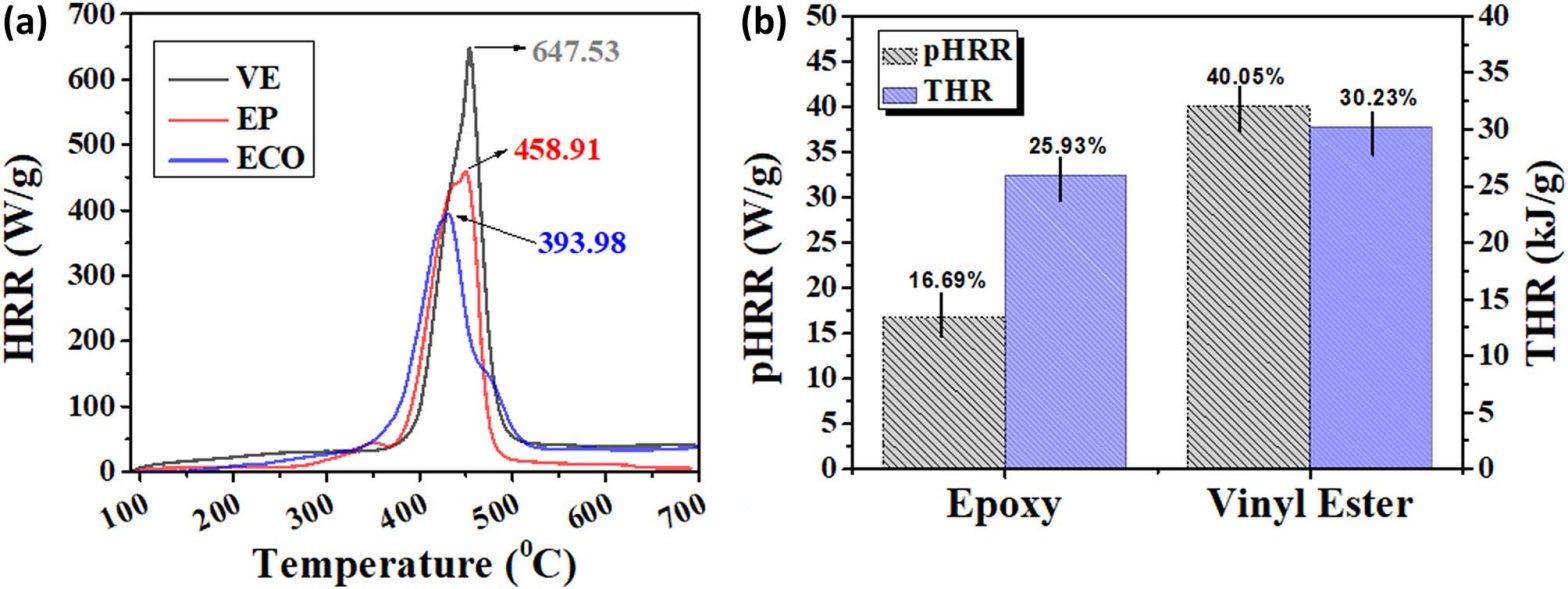
(**a**) HRR curves of ECO, vinyl ester, and epoxy; and (**b**) percent difference in flammability property for pHRR and THR.

In Fig. 9, microscale combustion calorimetry (MCC) shows the HRR, in particular the peak value of HRR (pHRR), which is considered to be the most important parameter in evaluating the fire safety of flame retardant materials^57^. It has the advantage of using small samples, and being a precise and extremely reproducible method, likewise it has been proven that the thermal combustion properties obtained by MCC are independent of the testing conditions^58–60^*.* The ECO exhibited a maximum pHRR of 373.3 W g^−1^ with a corresponding decomposition temperature of 431.763 °C and a THR of 24 kJ g^−1^, as shown in Table 6.

**Table 6:** Consolidated detailed data from microcombustion calorimetry.

	HRR (W/g)	pHRR (W/g)	Total HR (kJ/g)	Temperature (°C)	Heating rate (°C/s)	HRC Sum (J/g-K)	Peak Area (kJ/g)	N2 flow rate (cc/min)	O2 flow rate (cc/min)
Vinyl Ester	647.53	622.7	34.4	455.029	0.8877	624	34.44	79.966	19.982
Epoxy	458.91	448.1	32.4	450.634	0.8933	517	32.29	79.969	19.97
ECO	393.98	373.3	24	431.763	1.001	341	24.01	79.936	19.98

The two distinct peaks are shown in Fig. 9a, and the rate of heat release is known to be a two-step process wherein the first step pertains to the melting and degradation of the epoxides into tar and the second step indicates the combustion of the tar previously produced. In comparison with vinyl ester and epoxy in terms of pHRR and THR, significant changes were observed to confirm the improved flame retardant properties of the ECO. Figure 9b illustrates that an improvement of approximately 40.05% over vinyl ester and 16.69% over epoxy was measured from the ECO’s pHRR. Likewise, THR agrees with these results with a 30.23% and 25.93% increase over vinyl ester and epoxy, respectively.

The above results suggest the mechanism by which the hydroxyl function results in the increase in the flammability of the ECO, which potentially serves as a barrier to both mass and energy transport. The formation of more hydroxyl groups was confirmed through FTIR and ^1^H-NMR. Likewise, through the formation of a relatively uniform cross-linking network floccule layer, the flame retardant performance was achieved covering the entire sample surface, as confirmed through SEM and OM^61^.

The other data included in Table 6 depict the peak area, peak temperature, and heat release capacity (HRC), which follow the behaviours of pHHR and THR with ECO exhibiting a much lower value. These indicators support the idea that the flame was suppressed by the compositions of ECO in a shorter period compared to commercialised thermoset resins, even though the heating rate gradually increased and the char residue reported in TGA was much lower. The increase may be attributed to the changes of aliphatic compounds in ECO and an increase in hydroxyl, as observed in the spectral analysis.

In comparison, the ECO’s pHHR, 373.3 Wg^−1^, finding is an improvement above the recently published flammability property of bio-based materials defined by MCC. Menard et al. (2015) found that after curing with IPDA, phloroglucinol, a renewable resource used to make an epoxy monomer and phosphorus-containing reactive flame retardant (FR) coded as P3EP, produced a pHHR of 395 Wg^−1^ ^62^. Pourchet et al. (2019) investigated diepoxyisoeugenol phenylphosphate (DEpiEPP), a new green flame retardant copolymerized with glycidyl ether epoxy isoeugenol (GEEpiE), a completely bio-based polymer cured with anhydride hardener and with the addition of 2% phosphorus flame retardant additives, and found that it produced a 402 Wg^−1^ pHHR result^63^. Finally, Huang et al. (2021) looked at a bio-based monomer made from cardanol and cyclophosphazene (HECarCP) that was cured with 4,4′-diaminodiphenyl (DDM) and found a pHRR of 373.7 Wg^−1^ ^64^. As a result, our findings show that ECO is a viable bio-based flame retardant thermoset resin even without phosphorylation.

### Residual char analysis

**Figure 10:**
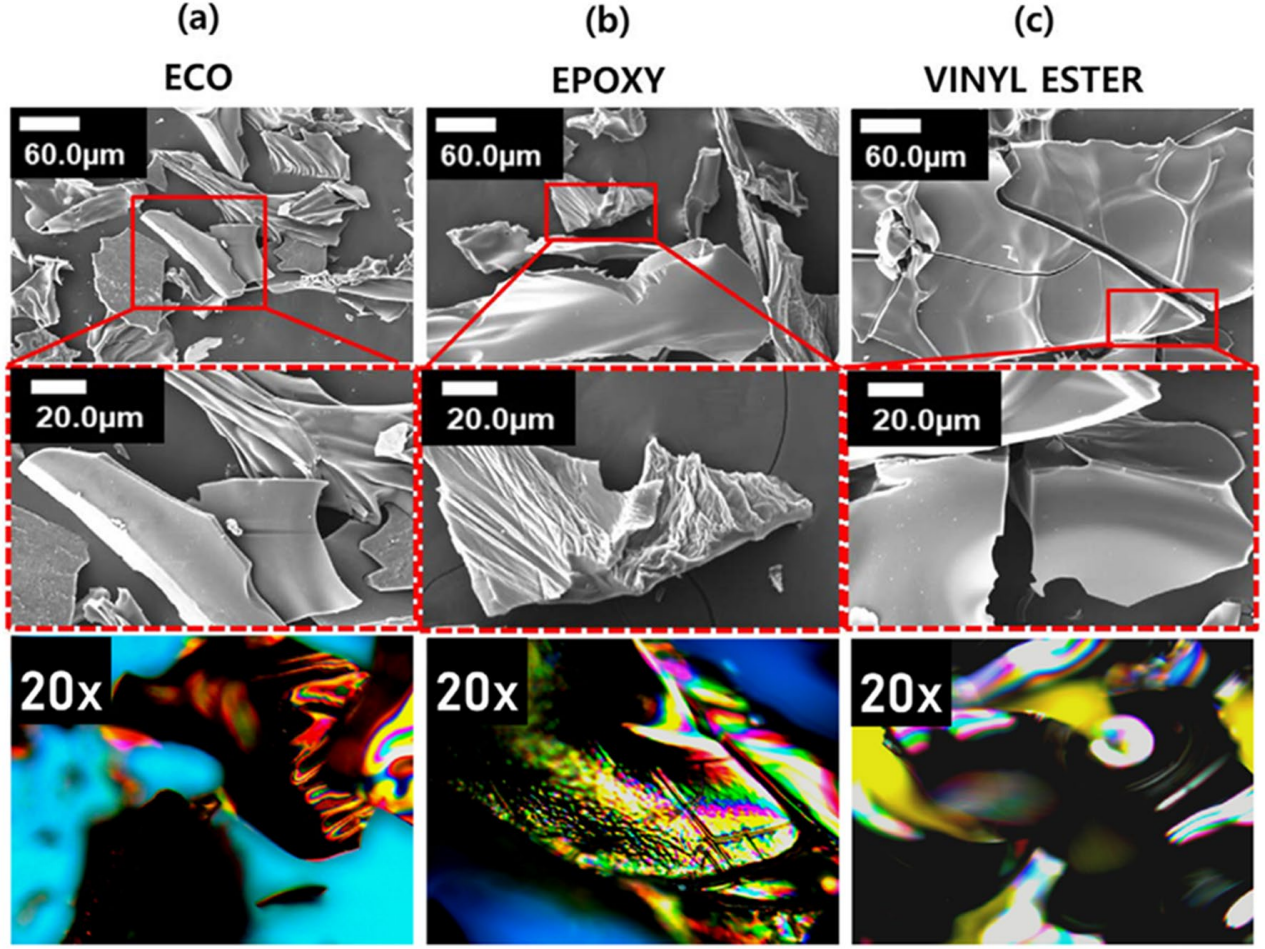
Morphological analysis of Residual Char using SEM and OM.

The morphologies of the residual chars produced from MCC tests are examined and compared with the residual chars of VE and EP to learn more about the flame-retardant mechanism of the non-phosphorylated ECO. The SEM and OM pictures of these residual chars are shown in Fig. 10, which show that the surface and contour have irregular-shape bulk. The ECO char linings, Fig. 10a, exhibit some thin to rough edges in SEM for scale bars 6 µm and 20 µm, but EP, Fig. 10b, and VE chars, Fig. 10c, are meant to show smooth and numerous surface ridges. The OM pictures at 20 × magnification of carbonaceous black chars for ECO and VE reveal a hazy color distribution due to its smooth surface, which allows the lights from the microscope to be seen. In EP, the ridges have a surface that is a combination of hazy and more vivid hues, indicating stiffness.

According to the residual chars, the char layer generated during combustion in ECO is more hard and compact. This structural structure promotes temperature gradients in the char layer while also protecting the matrix inside. Thermooxidative reaction of ECO moieties clearly improves char formation during burning, resulting in a protective char layer produced on the surface of thermosets serving as a barrier against heat and oxygen transport, and therefore the flame retardancy of the thermosets is considerably increased^65^.

**Figure 11:**
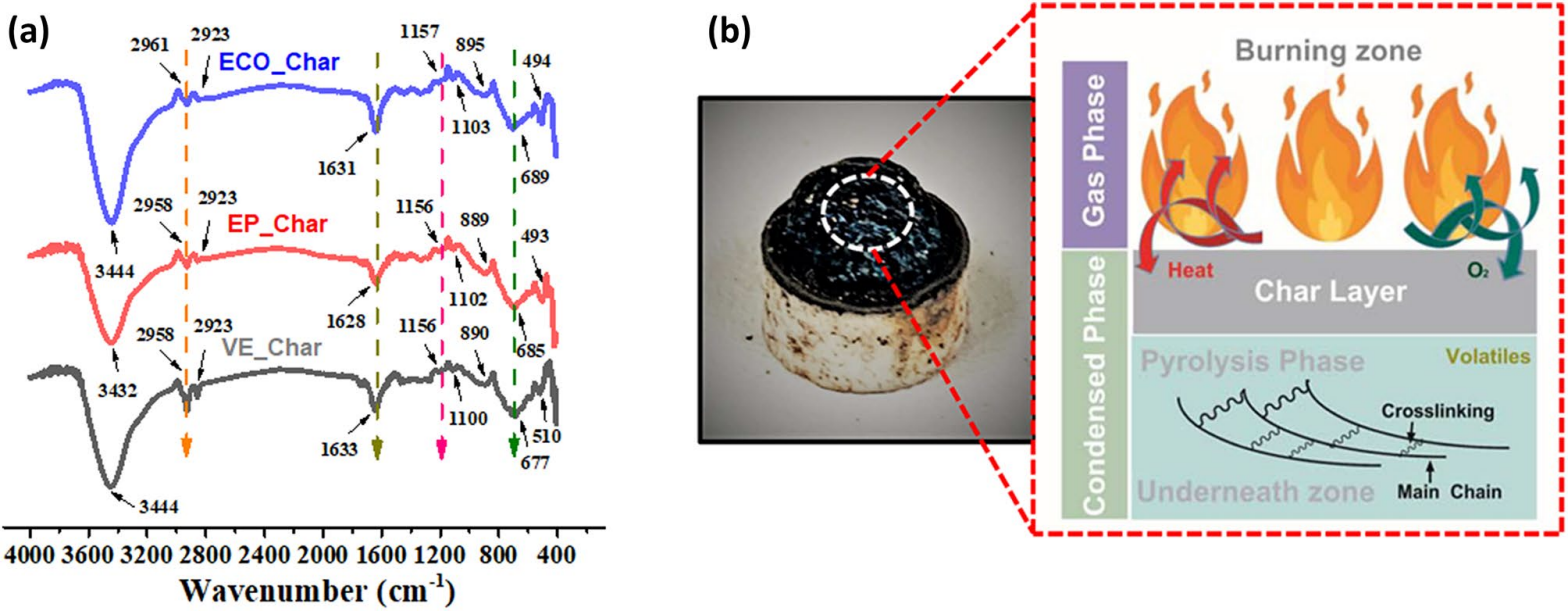
(**a**) Spectral analysis and (**b**) synergistic proposed schematic diagram of burning cycle of ECO thermoset resin.

The bands found in the spectra and the peak assignments are presented in Fig. 11a for further characterisation of residual char. Most of the peaks were detected in all samples, indicating that ECO does indeed act as a thermoset resin. In all samples, a wide OH peak was detected at 3432–3444 cm^−1^, indicating the existence of H bound OH. All organic materials exhibit peaks related to the C=O bond around 1628–1633 cm^−1^, with absorption frequencies indicating a ketone group for VE and EP and a residual C=C bond from ECO's lengthy aliphatic chain. Weak peaks at 895–890 cm^−1^ correspond to the stretching and bending modes of C=C–H. An examination of the FT-IR spectra reveals fascinating and substantial variations in the fingerprint region. Although modest, the peaks at 1103 cm^−1^, 895 cm^−1^, and 494 cm^−1^ for ECO, 1102 cm^−1^, 889 cm^−1^, and 493 cm^−1^ for EP, and 1100 cm^−1^, 890 cm^−1^, and 510 cm^−1^ for VE demonstrate that a distinct organic component is present. As a result, the previously disclosed SEM and OM pictures are confirmed^66^.

Figure 11b depicts a schematic diagram of the ECO's burning cycle for the flame retardant property. Heat is created as a result of the burning of volatile chemicals in polymeric materials such as ECO, and if enough heat returns to the polymer, its thermal breakdown is maintained owing to the self-sustaining of the burning cycle. This burning cycle may be disrupted, and the polymer combustion can be stopped, if some of the heat is removed from the burning cycle. By eliminating heat from the burning cycle, the pyrolysis rate of the polymer is reduced, and the combustion process is finally terminated. The char formed on the polymer surface not only acts as a smoke suppressant but also removes the heat of combustion. As a result, the type of flame retardant generally preferred for char formation is an intumescent flame retardant system, in which the charred layer restricts the diffusion of oxygen to the site of combustion and protects the underlying material from fire and heat flux. Thus, biobased thermoset resin is one of the best resources for this^67^.

## Conclusions

In this work, a novel bio-based epoxidised product from corn oil was successfully prepared under new parameter conditions using acetic acid (active oxygen carrier) and sulfuric acid (catalyst). The experimental results confirm that using a 1:10:0.9 molar ratio between double bonds, hydrogen peroxide, and acetic acid was an effective parameter condition compared to a much lower hydrogen peroxide concentration. An ECO of 91.7% of epoxidised corn oil was obtained under optimised reactive conditions of 50 °C, 4 h, and 1200 rpm. The FTIR, ^1^H-NMR, SEM, and OM confirmed the synthesis of a novel compound as a potential thermoset resin without using bisphenol-A and phosphorylation techniques. The appearance of new peaks, increase in energy bonding assessed using FTIR, and the confirmation of chemical shifts revealed through ^1^H-NMR strongly support the novelty of the ECO under the new design parameters. The SEM and OM morphologies supported and strengthened the above findings. Moreover, the significant reduction in pHRR and THR categorically suggests that ECO is a thermoset resin with excellent flammability properties comparable to those of two commercialised thermoset resins, epoxy and vinyl ester. Overall, the newly designed bio-based and non-phosphorylated ECO is a novel, efficient thermoset resin with potential value because of its flame retardancy, making it an attractive matrix for the composite and coating industries.
